# “When my Moods Drive Upward There Is Nothing I Can Do about It”: A Review of Extreme Appraisals of Internal States and the Bipolar Spectrum

**DOI:** 10.3389/fpsyg.2017.01235

**Published:** 2017-08-04

**Authors:** Rebecca E. Kelly, Alyson L. Dodd, Warren Mansell

**Affiliations:** ^1^Psychological Interventions Clinic for Outpatients with Psychosis, Maudsley Psychology Centre, South London and Maudsley NHS Foundation Trust London, United Kingdom; ^2^Department of Psychology, Northumbria University Newcastle-upon-Tyne, United Kingdom; ^3^School of Psychological Sciences, Faculty of Medical and Human Sciences, University of Manchester Manchester, United Kingdom

**Keywords:** bipolar disorder, mania, hypomanic personality, appraisals, emotion regulation

## Abstract

The integrative cognitive model provides a comprehensive account of bipolar disorder (BD) that, if empirically supported, has important potential implications for psychological therapies. This article is the first to review the evidence for this model. We evaluate the evidence (up to 2017) for four hypotheses derived uniquely from the model: extreme positive and negative appraisals of internal states are associated with (1) risk of developing BD; (2) BD diagnosis; (3) relevant clinical and functional outcomes including hypomanic and depressive mood symptoms; and (4) outcomes over time. Research involving individuals with diagnosed mood disorders as well as non-clinical populations is reviewed. The hypotheses were broadly supported and several consistent findings were not accounted for by alternative psychological models of BD. The evidence base is limited by a relative paucity of prospective studies; only 6 of the 31 studies identified. Implications for theory, research and clinical practice are discussed.

## Overview

In recent years there have been significant developments in our understanding of the psychological processes underlying bipolar disorder (BD). Psychological therapy is now recommended as a first-line treatment option for acute episodes and long-term relapse prevention (National Institute of Health and Care Excellence [NICE], 2014), alongside short-term and maintenance pharmacological treatments. However, there remains a need to develop and refine psychological models of BD and improve the effectiveness of psychological therapies. Historically, relapse prevention approaches that focus on identifying and coping with early warning signs have proved promising across several trials, with improvements in outcomes including hospitalisations, time to relapse and functioning; but these approaches have not always led to reductions in symptoms of depression and mania (Morriss et al., 2007). Cognitive-behavioural therapy (CBT) has also shown promise (Schöttle et al., 2011), but findings of some large trials have been mixed (e.g., Scott et al., 2006).

Naturalistic research indicates that patients receiving routine treatments (predominantly pharmacotherapy) remain highly symptomatic even outside episodes (Judd et al., 2002), and their problems extend more widely to include comorbidities that negatively affect outcome, such as anxiety (Otto et al., 2006) and trauma (Quarantini et al., 2010). Cognitive therapy approaches for BD typically draw on CBT approaches for depression, with additional psycho-education and behavioral strategies that aim to prevent future (hypo)manic episodes (e.g., Perlis et al., 2006). CBT for difficulties such as post-traumatic stress disorder and panic disorder seeks to directly address the specific cognitions and appraisals hypothesized to maintain distress as part of a vicious cycle of thoughts, feelings and behavior [negative appraisals of traumatic events and their sequalae (Ehlers and Clark, 2000) and catastrophic misinterpretations of bodily states (Clark, 1986), respectively]. In contrast, CBT for BD has not focused on specific appraisals that might maintain hypomania and mood instability. In order to more effectively address current symptoms and reduce relapse, psychological interventions need to be based upon empirically derived, well-specified models of the psychological processes underpinning the broad range of experiences that characterize BD. It is proposed that the Integrative Cognitive Model of BD (ICM; Mansell et al., 2007), represents one such model. The ICM was designed to integrate earlier psychological models, account for comorbidities and current symptoms, and explain the specific vulnerability to mania. This model has been tested in numerous studies over the past decade; it is therefore important and timely to synthesize and review this research to determine whether this model is sufficiently supported by research evidence for it to be used to inform and guide psychological therapy practice.

### Aims and Scope of Review

The aim of this review is to systematically summarize and evaluate the empirical evidence for four distinct predictions that follow from the ICM, pertaining to associations between extreme positive and negative appraisals of internal states and the bipolar spectrum. The ICM proposes that extreme appraisals are associated with the spectrum of difficulties characterized by mood swings, and can be both a vulnerability factor and part of a cognitive-behavioral maintenance cycle that exacerbates and perpetuates mood swing difficulties. Appraisals would be therefore expected to be associated with mania risk, non-clinical symptoms, diagnosed BDs, and mood-related outcomes over time.

We have utilized evidence from samples at risk of BD, as well as clinical samples, for a number of important reasons. First, the symptoms of BD can be assessed on a continuum within a non-clinical sample using clinically validated scales (e.g., Udachina and Mansell, 2007). Second, the measures of bipolar risk have been shown to be predictive of development of BD in long-term follow-up studies (e.g., Kwapil et al., 2000). Third, the majority of patients receive medication for their BD, whereas at-risk samples are typically medication-free and so the current and long-term use of medication cannot account for the findings in this group. Furthermore, results from analog studies are not confounded by a history of mood episodes. Nonetheless, evidence from clinical samples was considered as optimal. Evidence will be reviewed with respect to four theory-driven hypotheses:

(1) Extreme positive and negative appraisals of internal states will be positively associated with mania risk (both familial and behavioral).
(2) Extreme positive and negative appraisals will be associated with having a clinical diagnosis of BD and will be higher in this group than for diagnoses not commonly associated with mood instability (e.g., unipolar depression) or non-clinical controls.
(3) Extreme positive and negative appraisals will correlate with concurrent BD-relevant mood symptoms and experiences (e.g., depression and activation), well-being and personal functioning, in both clinical and non-clinical groups.
(4) Extreme positive and negative appraisals of internal states will predict the above outcomes over time, across the bipolar spectrum.

The ICM and its development are described in more detail below. Then, research evidence with regard to each of the hypotheses above is reviewed and the explanations of the findings within the ICM are contrasted with the alternative psychological models of BD. Finally, theoretical and therapeutic implications and important avenues for continued research efforts are discussed.

### Psychological Models of Bipolar Disorder

A number of psychological models of BD have been articulated that have added to our understanding of why symptoms of mania or depression may develop, escalate and persist (evidence for these models has been reviewed elsewhere, and so will not be described in detail here). However, we argue that existing models, such as the depression avoidance account (e.g., Abraham, 1911; Neale, 1988), the Behavioral Activation System dysregulation theory (e.g., Depue and Iacono, 1989; Urošević et al., 2008), and the circadian rhythm disturbance model (e.g., Healy and Williams, 1989; Jones, 2001) provide an incomplete account of the range of experiences within BD. There is a clear need for a psychological model and corresponding therapeutic approach that can address the full range of difficulties experienced by individuals with BD, including: symptoms of depression *and* mania including irritability and psychosis; comorbidity; mood lability and mood state switching; and mixed mood states. The Integrative Cognitive Model of BD (Mansell et al., 2007) proposes that extreme appraisals represent a psychological construct that can integrate disparate theoretical approaches to BD.

Drawing on the wider cognitive therapy literature, problematic appraisals of and responses to internal states and intrusions (e.g., one’s own mood and thoughts) are frequently highlighted as important psychological processes for determining the level and maintenance of distress and difficulty across a range of disorders [e.g., appraisals of voices as powerful and threatening in psychosis (Morrison, 2001); catastrophic misinterpretations of bodily sensations in panic disorder (Clark, 1986)]. Psychological models of BD have placed emphasis on extreme appraisals of internal experiences (i.e., the way in which individuals interpret events or stimuli and make sense of their experiences). However, different theories of BD have emphasized different types of appraisals. Within the depression avoidance theory, it is negative appraisals of depression, within BAS dysregulation theory it is reward and goal-related (positive) appraisals, and within models emphasizing circadian rhythm disturbance, it is self-referent appraisals of physiological changes such as increased energy. These models have previously been presented as distinct, if somewhat overlapping approaches, and have not considered that it might be specifically the combination of (or contradiction between) these different appraisals that might be driving mood instability.

### The Integrative Cognitive Model

**FIGURE 1:**
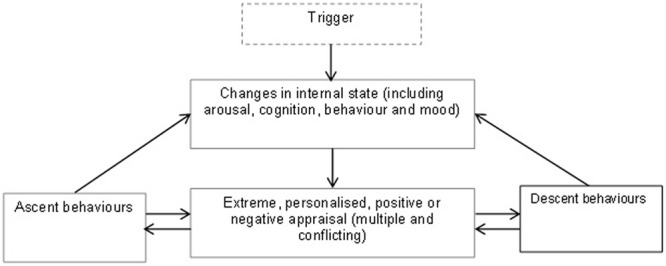
Simplified version of integrative cognitive model of bipolar disorder (BD) (adapted from Mansell et al., 2007).

The ICM (Mansell et al., 2007) proposes that extreme appraisals of internal states play a central role across the full spectrum of mood swing difficulties. Within the ICM, extreme appraisals of internal state change include interpretations of mood states (e.g., happy), physiological arousal (e.g., full of energy), observable behavior (e.g., talking more quickly), and cognition (e.g., racing thoughts). In contrast to other models of BD, both positive *and* negative appraisals of a range of current, past, or possible future experiences and their consequences are seen as pivotal. Specific kinds of appraisals are seen as especially pertinent to BD, including positive appraisals of high activation or (hypo)mania (e.g., “when I feel full of energy I am extremely funny and witty”); in addition to negative appraisals of these same mood states pertaining to potentially catastrophic consequences (e.g., “doing anything very active can lead me to have a breakdown”), self-criticism (e.g., “when I get overexcited, I am arrogant and overbearing”), and perceived loss or lack of control (e.g., “my high moods are outside of my control”). Extreme appraisals of low activation states such as tiredness and sadness are also argued to be relevant (e.g., “I cannot cope with being sad even for a short while”). The theory proposes that these extreme appraisals are maladaptive not because they are untrue (some individuals may have experienced these consequences of mood changes in the past themselves, or observed others experiencing them), but because individuals might appraise even minor, ambiguous changes in internal states (e.g., a brief increase in energy) in these extreme ways. Further, the theory proposes that appraisals contribute to mood dysregulation by prompting exaggerated, excessive and potentially contradictory attempts to exert control over, alter, or enhance internal states (see **Figure 1**). These behaviors and emotion regulation efforts may become self-fulfilling by leading to further internal state changes and contributing to a ‘vicious cycle’ that exacerbates and maintains symptoms (Mansell et al., 2007).

More specifically, for example, an individual may appraise their thoughts racing (a high activation state) as both a sign of their great intelligence and as a sign that they are losing control of their mind. They may then alternate between trying to speed up their thinking further (e.g., taking stimulants) and trying to slow down their thoughts (e.g., social withdrawal). Conflicting positive and negative appraisals of the same states are therefore proposed to explain mood fluctuations and instability, an aspect of BD that the other models struggle to account for.

### Measurement of Appraisals

Appraisals of internal states relevant to the bipolar spectrum have been primarily assessed using self-report tools. The Hypomanic Attitudes and Positive Predictions Inventory (HAPPI; Mansell, 2006) was developed in tandem with the ICM, and therefore assesses both positive and negative appraisals of high and low activation internal states. The initial 104 item inventory was developed through clinical observation and reviewing relevant therapy manuals, and was reduced to a 50-item version comprising the items which best distinguished individuals with BD from non-clinical controls. This version incorporated five themes that reflect the subtypes of appraisal described earlier (Mansell, 2006). An expanded 61-item version added appraisals of activated states as prompting self-criticism, and being confusing and overwhelming (Dodd et al., 2010). Further studies have used abridged versions (Mansell and Jones, 2006; Fisk et al., 2015). Factor analyses have suggested at least five distinct subsets of extreme positive and negative appraisals of internal states are assessed by the HAPPI, including catastrophic appraisals of activated states and appraisals relating to anticipated success when activated (e.g., Mansell et al., 2008; Dodd et al., 2010, 2011a).

The remaining scales we include in the review assess appraisals of internal states but they were not designed with the ICM in mind and so they do not assess the combination of positive and negative appraisals of the same internal states. The Hypomania Interpretations Questionnaire (HIQ; Jones et al., 2006) is based on the appraisals of circadian rhythm disturbance theory and so it specifically measures positive self-referent appraisals of hypomania-relevant experiences, such as “If I woke up earlier than normal and felt full of energy, I would probably think it was because I am a happy, positive and energetic person”, versus normalizing explanations (“Something has disrupted my routine”). The HIQ does not assess negative appraisals of these states. The corresponding Interpretations of Depression Questionnaire (IDQ; Jones and Day, 2008) specifically assesses negative appraisals of depression-relevant experiences. Appraisals of mood swings; the overall changeability of mood rather than a specific type of internal state; have been assessed using a version of the Brief Illness Perceptions Questionnaire specifically adapted for BD (BIPQ; Lobban et al., 2013). The BIPQ-BD items refer to beliefs about, and interpretations of, mood swings (e.g., “how concerned are you by your mood swings?”). The types of cognitions assessed by this measure are derived from the Self-Regulation Model (SRM; Leventhal et al., 1984), which proposes that beliefs about illnesses predict health outcomes. Whilst this is not a theory of BD specifically, but of illnesses in general, studies utilizing this scale were included because the scale items assess interpretations of mood swings.

Importantly, each of the measures listed above assess appraisals of, and beliefs about, internal states that are ambiguous, potentially innocuous and may be experienced by patients and the general population alike (e.g., excitement, energy levels increasing, or feeling sad for a short while), and not internal states that are always clinically significant, or diagnostic markers for BD (e.g., flight of ideas, or persistent decreased pleasure).

## Method

### Search Strategy

A search was conducted using two large academic databases (Web of Science and PubMed-MEDLINE), from the beginning point of each database through the middle of 2017, using preselected keyword terms. The following search string was used: (appraisals OR cognit^∗^ OR beliefs OR interpretations OR perceptions) AND (internal states OR affect OR mood OR activation) AND (mania OR hypomania OR hypomanic personality OR BD). Reference lists of the obtained articles were reviewed for relevant articles, and searches were performed for well-known measures of extreme appraisals.

**FIGURE 2:**
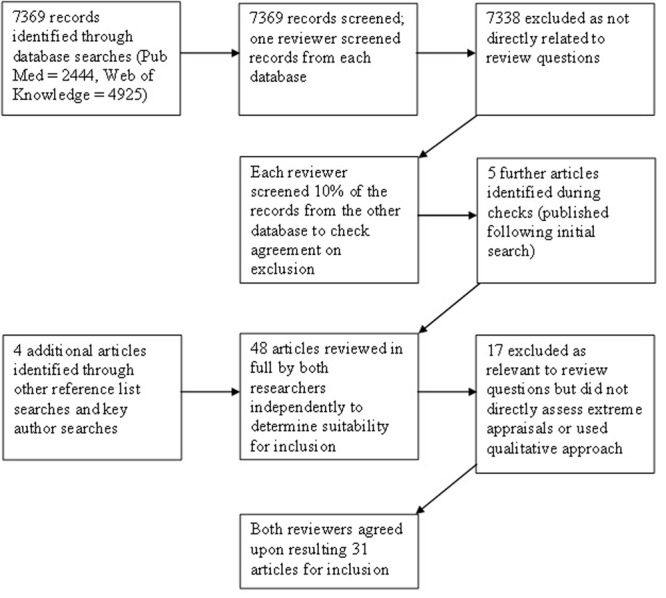
Article search flowchart.

The two lead authors conducted independent searches and screened articles from one of the two databases. As a reliability check for agreement, 10% of articles identified in each database were screened by the other author. Titles and abstracts identified as potentially meeting inclusion criteria were then reviewed in detail by both researchers. Only those articles agreed upon by both reviewers were included (see **Figure 2**).

### Inclusion Criteria

All studies had to include a quantitative measure of extreme appraisals of internal states relevant to BD. A range of outcomes were considered relevant to supporting or disconfirming the hypotheses derived from the model, including clinical BD diagnosis, relevant symptoms such as depression and activation, and risk for mania. The following types of studies were therefore eligible for inclusion: (1) Studies that included a measure of either familial risk for BD (i.e., parental mood disorder diagnosis) or elevated scores on measures of behavioral risk. Behavioral risk indices include: The Hypomanic Personality Scale (Eckblad and Chapman, 1986), a commonly used measure that assesses trait extroversion and mood lability and predicts future BD diagnosis (Kwapil et al., 2000); the Mood Disorders Questionnaire (MDQ; Hirschfeld et al., 2000), which establishes ‘caseness’ using a combination of history of manic symptoms as well as their co-occurrence and severity; and the General Behavior Inventory (Depue et al., 1981), which determines risk for BD and unipolar depression based on history of relevant experiences. (2) Studies comparing individuals with diagnoses of BD, as determined by previous diagnosis or structured clinical interview, to other clinical or non-clinical control groups. (3) Studies measuring bipolar-relevant mood symptoms and experiences across the bipolar spectrum (diagnosed and non-clinical samples), including (hypo)manic symptoms (e.g., activation) and depression and either their relationship to appraisals or change in these outcomes following therapy for BD specifically targeting and assessing extreme appraisals.

### Exclusion Criteria

(1) Studies not published in English, (2) Studies not published in a peer-reviewed outlet, and (3) Studies not reporting empirical evidence (e.g., theoretical and review articles).

### Quality Assessment

The minimum criteria for inclusion were quantitative measurement of extreme appraisals using a validated measure with adequate psychometric properties, and valid and reliable measures used to assess mood symptoms, further clinical and functional outcomes, and mania risk.

### Screening

A total of 31 relevant articles were identified (28 quantitative studies and a case series evaluating a therapeutic approach). These studies varied in their methodology and sampled a range of populations, including non-clinical and at-risk samples (Supplementary Table S1) and clinical samples (Supplementary Table S2). We have focused on findings specifically pertaining to appraisals of internal states. Where associations between other psychological constructs and outcomes were analyzed separately to appraisals of internal states, findings have not been reported. However, where other psychological processes have been analyzed as a potential covariate in the same model as extreme appraisals, and the incremental validity and unique contribution of appraisals to outcomes can therefore be ascertained, these findings are reported.

## Synthesis of Findings

### Extreme Positive and Negative Appraisals Are Associated with Mania Risk

#### Familial Risk

Three cross-sectional studies (see Supplementary Table S1) have explored endorsement of extreme appraisals of internal states in children of parents with BD (CPB), one using the HIQ (Espie et al., 2012) and two using the HAPPI (Pavlickova et al., 2014; Ruggero et al., 2015). All studies assessed the diagnostic status of parents as well as the offspring, allowing for the exploration of the role of familial risk alone versus personal experience of BD. Espie et al. (2012) found that adolescent CPB did not score more highly on positive, self-referent appraisals of hypomanic states. Pavlickova et al. (2014) found that CPB did not endorse more positive and negative appraisals of internal states than adolescents with no parental BD; interestingly and perhaps surprisingly this study found no associations between parental mood diagnosis and numerous psychological measures previously found to be associated with BD. Pavlickova et al. (2014) did, however, identify an interaction effect, such that adolescents who had a parent with BD and themselves met criteria for any psychiatric disorder scored significantly higher on the HAPPI than both unaffected offspring of parents with BD and unaffected controls. Ruggero et al. (2015) found that extreme appraisals of internal states were elevated among adult CPB relative to those whose parents had no mood disorder, and this association remained significant when controlling for their own mood diagnosis.

Of the studies of familial risk and extreme appraisals, only Ruggero et al. (2015) looked in detail at the subscales of the HAPPI and found evidence for elevation in both negative and positive appraisals. Offspring of parents with any mood disorder differed from controls on appraisals of high mood as being necessary to avoid failure, as well as appraisals of high mood as a sign of losing control. Appraisals about the need to maintain personal autonomy were also higher among the two familial risk groups. Social self-criticism and grandiose appraisals specifically were elevated in CPB compared to controls. When controlling for offspring diagnosis, findings were largely corroborated. All of these studies had relatively small sample sizes, particularly when splitting parent and offspring groups into diagnostic sub-samples.

#### Behavioral High-Risk for BD

Several studies have examined cross-sectional associations between appraisals and mania risk (see Supplementary Table S1). Jones et al. (2006) found the HIQ was positively associated with mania risk, and more variance was explained by positive self-appraisals than dysfunctional attitudes. This has been replicated across several studies (Jones and Day, 2008; Johnson and Jones, 2009; Dempsey et al., 2011). Negative self-appraisals of depression-relevant experiences (IDQ; Jones and Day, 2008) were modestly associated with mania risk, but uniquely contributed only to the variance in depression (Jones and Day, 2008; Dempsey et al., 2011; for more detail, see Extreme Positive and Negative Appraisals of Internal States Are Associated with BD-Relevant Symptoms and Experiences). Principal components analysis (Dempsey et al., 2011) found considerable conceptual overlap between the HIQ and response styles such as amplifying positive affect, and between the IDQ and response styles such as dampening positive affect and ruminating on low mood. Positive, but not negative, cognitive styles predicted mania risk. However, Johnson and Jones (2009) found that items on the HIQ were separable from overconfidence, impulsivity, and ruminative response styles; overly positive appraisals were significantly associated with mania risk. Using the HAPPI, there are again associations between extreme appraisals and mania risk (Fisk et al., 2015; Haigh and Dodd, 2017). Other studies have considered the HAPPI and mania risk (hypomanic personality) as independent variables when investigating associations with mood, rather than reporting associations between extreme appraisals and hypomanic personality (Mansell et al., 2008; Dodd et al., 2010, 2011b; see Extreme Positive and Negative Appraisals of Internal States Are Associated with BD-Relevant Symptoms and Experiences).

After grouping participants into mania risk and controls, Ankers and Jones (2009) found higher positive self-appraisal scores in the mania risk group. When selecting participants on the basis of their scores on the HAPPI measure of multiple, conflicting positive and negative appraisals of different internal states, those participants scoring in the 90th percentile had elevated hypomanic personality scores (Dodd et al., 2013) directly supporting the hypothesis. Positive appraisals of hypomanic states alone, as measured by the HIQ, were not significantly elevated in this group. One study has used an alternative behavioral high-risk measure. Dodd et al. (2011c) split students into the following groups, using established criteria for the General Behavior Inventory (Depue et al., 1981); mania risk, depression risk, and controls. Using a computerized task based on the HAPPI, positive and negative appraisals of the same internal states (high and low activation) were directly and systematically assessed. Findings suggested a more pervasive negative cognitive style among those at risk of depression, who endorsed more negative appraisals of all internal states than positive appraisals. Individuals at risk for mania endorsed more positive appraisals of high activation states. Group comparisons indicated that both the depression- and mania-risk groups were more likely to endorse catastrophic appraisals of low activation than controls.

One study explored appraisals of internal states and mania risk among adolescents aged 14–15 years, using an adapted, age-specific version of the HAPPI (Kelly et al., 2016). Adolescents who scored highly on both positive and negative appraisals of activated internal states were at increased risk of BD, as assessed by the Mood Disorders Questionnaire (MDQ; Hirschfeld et al., 2000).

#### Summary of Findings

The findings of those studies examining familial risk and appraisal style are somewhat inconsistent: one study found no psychological indices to differ between CPB and controls, two studies found that appraisals as assessed by the HIQ and dysfunctional attitudes did not differ between CPB and controls, but one study found differences on HAPPI appraisals between CPB and controls that were maintained when controlling for whether the offspring themselves had a mood disorder diagnosis. This inconsistency could be explained by the combined positive and negative appraisals assessed by the HAPPI better distinguishing those at risk for BD, or alternatively by small effect sizes. In contrast, positive appraisals of hypomanic states were consistently related to behavioral-high risk. Findings for negative appraisals of low mood were more mixed; they were not uniquely associated with mania risk. This specifically goes against the manic-defense hypothesis, which hypothesizes that mania is driven by negative appraisals of depressed and low energy states.

Consistent with the ICM, only extreme positive and negative appraisals, and not normalizing appraisals, were heightened in people at familial or behavioral high risk of developing mania (Jones et al., 2006; Jones and Day, 2008; Ankers and Jones, 2009; Johnson and Jones, 2009; Dempsey et al., 2011; Espie et al., 2012). Findings across risk measures provide more consistent support for the ICM than the BAS and circadian disturbance appraisal models (which only emphasize positive appraisals of hypomanic states), because whilst there was some inconsistency in terms of whether positive appraisals were related to bipolar risk (Jones et al., 2006; Ankers and Jones, 2009; Dodd et al., 2013), where positive and negative appraisals of activated internal states were assessed together they were predictive (Dodd et al., 2011c, 2013).

### Extreme Positive and Negative Appraisals Are Associated with a Diagnosis of BD

In group studies comparing individuals with BD to other groups, individuals with BD consistently scored more highly on positive and negative appraisals of internal states compared to non-clinical controls (Mansell, 2006; Alatiq et al., 2010; Mansell et al., 2011; Pavlickova et al., 2014; Ruggero et al., 2015; Tosun et al., 2015). Both extreme positive and negative appraisals (HAPPI) and positive self-referent appraisals of hypomanic states (HIQ) accurately predicted BD diagnosis (Jones et al., 2006; Mansell and Jones, 2006; Mackali et al., 2014), controlling for current mood. The HAPPI also discriminated individuals with BD from those with unipolar depression (Alatiq et al., 2010; Mansell et al., 2011), supporting the ICM proposition that extreme positive and negative appraisals of internal states are specifically related to BD. Normalizing appraisals did not differentiate individuals with BD from control groups (Jones et al., 2006; Mansell and Jones, 2006).

Mansell (2006) found that those with BD scored significantly higher than non-clinical controls on every HAPPI subscale, confirming that both positive and negative appraisals are elevated. Alatiq et al. (2010) reported that those with BD had significantly higher scores than the control group *and* the group with unipolar depression specifically on appraisals relating to perceived negative perceptions of the self by others, and about how to respond to activated internal states (e.g., that they must keep generating more ideas); and differed from controls on catastrophic appraisals of activated states. When examining the factors identified by Mansell et al. (2008, 2011) reported a pattern of findings (see Supplementary Table S2) suggesting that appraisals of activated mood as entailing imminent success (positive) and a lack of personal control over mood (negative) were pertinent across the BD spectrum including ‘softer’ manifestations; they distinguished both recently relapsed and recovered BD groups, as well as those with a history of hypomania only, from non-clinical controls. Interestingly, the hypomania group did not differ significantly from those with BD on total extreme appraisals scores, but had significantly lower scores than the *relapsing* BD group on catastrophic appraisals; tentatively suggesting that catastrophising may play a key role in the recurrence of mood episodes. The recently relapsed BD group differed from non-clinical controls on all subscales. These findings together provide unique support for the ICM, the only model of BD that emphasizes the detrimental role of negative appraisals of activated mood states.

Ruggero et al. (2015) also found that those with BD scored higher on every (positive and negative) appraisal subscale (from Dodd et al., 2011d) compared to those with unipolar depression and controls (differences by parent diagnosis are reported in Extreme Positive and Negative Appraisals Are Associated with Mania Risk section). The sample size for the BD group in these comparisons was small, and findings must be treated tentatively. BD diagnosis, but not unipolar depression, was associated with all types of appraisal except Success Activation and Grandiose Appraisals, controlling for parent’s diagnosis, again emphasizing it is not simply extreme positive appraisals that distinguish BD. Appraisals related to critical thoughts about the self and perceived criticism from others were associated with own diagnosis of BD but not parental diagnosis, suggesting these appraisals specifically may be associated with experience of, and not risk for, BD.

Kelly et al. (2011) combined data from a number of studies (Mansell, 2006; Alatiq et al., 2010; Dodd et al., 2011a; Mansell et al., 2011) to compare individuals with BD to individuals with unipolar depression and controls, specifically selecting HAPPI items describing positive and negative appraisals of high or activated mood states to directly test the hypothesis. Individuals with BD tended to appraise activated mood states in both extremely positive and extremely negative ways. Interestingly, individuals with unipolar depression and controls also appraised activated states in positive ways, albeit to a lesser extent, but individuals with BD were distinguished by making both extremely positive and extremely negative appraisals of activated states.

#### Summary of Findings

The finding that extreme, self-relevant appraisals are heightened among individuals with a self-reported diagnosis of BD (Jones et al., 2006; Mansell, 2006) has been replicated in those with a confirmed research diagnosis using semi-structured clinical interviews (e.g., Alatiq et al., 2010; Kelly et al., 2011; Pavlickova et al., 2014; Ruggero et al., 2015), for both the HAPPI and HIQ. Where measured together, the HAPPI and HIQ were correlated, but made unique contributions to differentiating those with BD from controls (Jones et al., 2006). Studies have utilized different theoretical or factor analytically derived subsets of appraisals and it is therefore difficult to draw overall conclusions about the types of appraisals most closely associated with BD. However, multiple studies found that the presence of negative or contradictory appraisals of activated mood states characterized BD groups. Further, total scores on the HAPPI measure, an index of the extremity of both positive and negative appraisals, was more consistently related to BD than individual subscale scores, suggesting that perhaps what uniquely characterizes BD is appraisals that are multiple and conflicting. This is not consistent with models of BD emphasizing overly positive cognitive styles, but provides specific support for the ICM.

### Extreme Positive and Negative Appraisals of Internal States Are Associated with BD-Relevant Symptoms and Experiences

#### Non-clinical Populations

Nine studies explored cross-sectional associations between extreme appraisals and mood symptoms, predominantly using student samples (Jones and Day, 2008; Mansell et al., 2008; Dodd et al., 2010, 2011a, 2013; Dempsey et al., 2011; Kelly et al., 2011, 2016; Tosun et al., 2015; Dodd and Haigh, 2017; Haigh and Dodd, 2017). Extreme appraisals of internal states were consistently positively associated with activated mood (Jones et al., 2006; Jones and Day, 2008; Dodd et al., 2011a, 2013; Kelly et al., 2011; Dodd and Haigh, 2017; Haigh and Dodd, 2017). Those scoring at the 90th percentile of the HAPPI had significantly more self-reported activation (hypomania) and observer-rated activated behaviors synonymous with pressure of speech (Dodd et al., 2013). These studies utilized the Internal States Scale (ISS; Bauer et al., 1991), which has been widely used to measure analog hypomania and depression symptoms.

The combination of positive and negative appraisals as assessed by the HAPPI has also been related to self-reported history of hypomanic symptoms (MDQ; Hirschfeld et al., 2000) in five non-clinical samples (Mansell et al., 2008; Dodd et al., 2013; Mackali et al., 2014; Tosun et al., 2015; Kelly et al., 2016). However, Fisk et al. (2015) report a null finding such that an abridged version of the HAPPI was not uniquely associated with manic symptoms on the Altman Rating Scale for Mania (Altman et al., 1997), which measures a fuller range of manic symptoms than the ISS (Bauer et al., 1991).

Kelly et al. (2012) combined data from a number of analog studies to examine specific predictors of high and low mood symptoms in a non-clinical sample. Activation was uniquely associated with positive appraisals of hypomanic, energized states, whilst negative appraisals of the same states were uniquely associated with depressive symptoms. Kelly et al. (2016) replicated these findings in an adolescent sample aged 14–15, and also found that positive appraisals of hypomanic states were uniquely associated with irritability. In another study (Dodd et al., 2011a), only Social Self Criticism was significantly associated with activation, while Mansell et al. (2008), Haigh and Dodd (2017) report that all of the HAPPI subscales were related to activation (with catastrophic appraisals having a negative association in the latter).

As would be expected, negative self-appraisals of low mood (IDQ; Jones and Day, 2008) were associated with depression (Jones and Day, 2008; Dempsey et al., 2011). Findings regarding depression and other forms of extreme appraisals are inconsistent (Jones and Day, 2008; Fisk et al., 2015; Dodd and Haigh, 2017; Haigh and Dodd, 2017), although depression was elevated in a group with higher HAPPI scores (Dodd et al., 2013) and negative appraisals of activated states were associated with depression (Kelly et al., 2012). Looking at the different subscales derived from versions of the HAPPI, the pattern of appraisal types associated with depression varies across the non-clinical studies (Mansell et al., 2008; Dodd et al., 2011a; Haigh and Dodd, 2017; see Supplementary Table S1 for detail).

#### Clinical Populations

Three studies investigated cross-sectional associations between appraisals and outcomes in a clinical sample (see Supplementary Table S2). Appraisals were associated with length of remission, such that those who had been in remission longer endorsed fewer extreme and conflicting appraisals of internal states (Tosun et al., 2015). Appraisals were positively associated with a lifetime history of manic symptoms and current depressive (but not manic) symptoms (Lobban et al., 2013; Mackali et al., 2014).

Where reported, normalizing appraisals were not associated with mood symptoms (Jones and Day, 2008; Dodd et al., 2011d). There was one exception (Dempsey et al., 2011) that reported small but significant relationships between depression and normalizing appraisals of hypomanic experiences (positive), and between well-being and normalizing appraisals of depression (negative).

#### Summary of Findings

Across clinical and non-clinical samples, extreme appraisals of internal states are typically correlated with concurrent mood symptoms, in particular activation symptoms, often when controlling for potentially confounding psychological variables. Extreme appraisals of internal states were also associated with self-reported past hypomania and mania experiences in non-clinical samples, and with length of remission in a clinical group. Specific kinds of appraisals were related to specific kinds of symptoms. Negative appraisals of both activated and low mood states related to depression. However, both positive and negative appraisals of activated states tended to be associated with concurrent activation/ hypomania symptoms, consistent with the ICM, and normalizing appraisals of hypomanic experiences were also associated with depression. This would suggest that these findings are *not* simply reflective of a general mood-congruent perceptual bias, whereby individuals who are depressed endorse negative statements and individuals who are hypomanic endorse positive statements.

### Extreme Positive and Negative Appraisals Predict BD-Relevant Symptoms and Experiences Prospectively

#### Non-clinical Populations

Two prospective studies have investigated relationships between extreme appraisals of internal states and mood symptoms in non-clinical populations. Dodd et al. (2010) found that positive and negative appraisals as assessed by the HAPPI predicted activation and depression 3 months later, controlling for baseline symptoms. This measure independently predicted activation symptoms when controlling for reward sensitivity and hypomanic personality, consistent with the ICM proposal that extreme positive and negative appraisals make a unique contribution to BD, alongside other psychosocial variables. Dodd et al. (2011b) asked students to complete the ISS (Bauer et al., 1991) as well as ascent and normalizing behaviors twice daily for 4 consecutive days. Extreme positive and negative appraisals independently predicted activation and depression, as well as ascent behaviors, over this period, again when controlling for reward sensitivity, hypomanic personality, and baseline symptoms. Extreme appraisals did not predict the use of mood-balancing ‘normalizing’ behaviors, indicating that individuals who appraise internal states in extreme and personalized ways tend to engage in activation-enhancing ascent behaviors, but not adaptive, normalizing behaviors. The HAPPI measure used here incorporates both appraisals of high moods as leading to success or avoidance of failure (e.g., “…I can bring about a large rise in my social status,” “unless I am active… I will end up a failure”) and appraisals relating to discounting potentially negative consequences (e.g., “…my fears and worries are no longer real,” “…everything will work out perfectly”), suggesting that appraisals might impact upon behavior due to either a goal-oriented motivation to maintain or enhance the high, or to impulsive responses to high mood, or both.

#### Clinical Populations

Four prospective studies of clinical groups investigated associations between extreme appraisals or beliefs at baseline and symptoms and/or functional outcomes at follow-up (Dodd et al., 2011d; Lobban et al., 2013; Fletcher et al., 2014; Palmier-Claus et al., 2015). In each study, diagnosis of BD was confirmed using a semi-structured clinical interview. Dodd et al. (2011d) reported that baseline extreme positive and negative appraisals as assessed by the HAPPI predicted symptoms of activation and conflict (reflecting irritability and mood lability) after 4 weeks. Notably, the HAPPI predicted subsequent symptoms even when controlling for relevant clinical variables, including baseline symptoms, number of months since previous mood episode, and number of hours of CBT previously undertaken. Clinical variables such as number of previous episodes, age of onset, and months since last hospitalization were not correlated with activation or interpersonal conflict symptoms. Regression analyses indicated that the subscale of appraisals relating to ‘increasing activation to avoid failure’ specifically predicted activation symptoms, whilst the other HAPPI subscales, and the appraisals assessed by the HIQ did not (see Supplementary Table S2).

In a different analysis of this data (Palmier-Claus et al., 2015), positive and negative appraisals (as defined by Kelly et al., 2011) were explored separately using multi-level regression. Positive appraisals directly related to subsequent activation (partially mediated by ascent behaviors). Negative appraisals were not associated with depression or descent behaviors – yet descent behaviors directly predicted depression. This pattern is to be expected; this study specifically examined appraisals of activated (high mood states) and not appraisals of low mood states. However, the descent behaviors assessed represented negative, ruminative responses to low mood analogous to those shown to be related to depression (e.g., Nolen-Hoeksema and Morrow, 1993). Thus, the findings relating to hypomania symptoms are highly consistent with the ICM in showing that extreme interpretations of, and responses to, a particular mood state seem to lead to further experiences of the same mood state. Findings related to depression here are consistent with both the ICM and other models such as the Response Styles Theory of depression (Nolen-Hoeksema et al., 1998).

Lobban et al. (2013) found beliefs about mood swings influenced relapse risk, depression and functioning over 24 weeks, even when controlling for baseline symptoms, current mood stabilizer use, and number of previous episodes; however, there were no associations with mania (see Supplementary Table S2 for details). Fletcher et al. (2014) found that the HAPPI was associated with variability in depression over 6 months but had no association with self-reported hypomania, or clinician-rated depressive or manic episodes, at follow up.

#### Summary of Findings

Across clinical and non-clinical samples, there is evidence that extreme appraisals of internal states predict bipolar-relevant symptoms, including activation, irritability (conflict) and depression, over time, often when controlling for potentially confounding clinical and psychological variables. Variation in measures of appraisals and symptoms make it challenging to draw firm conclusions about the specific types of appraisals that relate to outcomes over time. The total HAPPI score, which provides an overall measure of extremity of and conflict between positive and negative appraisals, predicted mood over time, but when looking at subscales it was specifically appraisals about high mood enabling the avoidance of failure that related to hypomania 4 weeks later (Dodd et al., 2011d). Different types of appraisals might relate to outcomes over differing time intervals. For example, individuals seeking to drive their mood up to avoid failure and achieve goals might be expected to increase their goal-pursuit behavior, which might lead to short-term increases in activation and hypomania. Appraisals and beliefs relating to a loss of control over mood appeared relevant to depression across studies (Dodd et al., 2011d; Lobban et al., 2013). However, no significant associations were found between self-reported appraisals and observer-rated (hypo)mania where assessed (Lobban et al., 2013; Fletcher et al., 2014).

#### Investigations of Psychological Therapy

Searson et al. (2012) reported on a case series of CBT focusing on identifying and modifying extreme appraisals of internal states, which included the HAPPI as a measure for formulation and assessing mechanism of change. There were reductions in mood symptoms and improvements in functioning at the end of treatment, with particularly large effects for depression. These effects were maintained at 1-month follow-up. This study also found that overall HAPPI scores significantly decreased from pre to post therapy; however, mediational models were not tested. This type of modeling is important to determine the mechanism of change in therapy; if appraisals mediated change in outcomes, this would provide good support for the theory that they play a significant role in mood dysregulation.

## Discussion

### Evaluating Results in Relation to the ICM

This review investigated theory-driven hypotheses generated from the ICM (Mansell et al., 2007). We predicted that extreme positive and negative appraisals of internal states would be: (1) associated with indices of mania risk, (2) elevated in individuals with a diagnosis of BD relative to other clinical and non-clinical groups, (3) associated with outcomes such as mood symptoms and functioning, and (4) associated with prospective outcomes. The findings broadly support all hypotheses, in the context of a number of qualifications and limitations.

Consistent with hypotheses 1 and 2, associations between appraisals and behavioral high-risk and diagnosis of BD were generally robust across studies and measures of appraisal. Familial risk studies indicated appraisals were associated with personal bipolar experiences, or an interaction between parental and own mood disorder diagnosis; only one study demonstrated an independent association between parental BD and appraisals when controlling for the offspring’s own diagnosis (Espie et al., 2012; Pavlickova et al., 2014; Ruggero et al., 2015). Not all types of extreme appraisals differentiated those at risk or with BD from controls. It seems intuitive that the relative importance of some appraisal types will differ between groups. For example, appraisals of activated states as signaling imminent catastrophe or loss of control would be expected to be more pertinent among those who have had full-blown manic episodes, and therefore particularly pertinent for BD compared to unipolar depression and non-clinical groups, and findings are consistent with this (Mansell, 2006; Alatiq et al., 2010; Mansell et al., 2011; Ruggero et al., 2015). These appraisals seem to show a dose-response relationship with the continuum of BD; they correlate with mood symptoms, and are most elevated in individuals with BD, followed by individuals who have experienced hypomania, followed by other groups.

In line with the third and fourth hypotheses, cross-sectional and longitudinal evidence indicated that appraisals were associated with BD-relevant outcomes, such as mood and functioning. These relationships were independent of other relevant psychological processes, where controlled for. Relationships persisted in non-clinical samples when controlling for reward sensitivity (Jones and Day, 2008; Mansell et al., 2008; Dodd et al., 2010, 2011b, 2013), response styles (Jones and Day, 2008; Johnson and Jones, 2009; Dempsey et al., 2011; Fisk et al., 2015; Kelly et al., 2016), reasoning bias (Haigh and Dodd, 2017); and dysfunctional attitudes (Jones and Day, 2008; Dodd et al., 2010). Dysfunctional attitudes did not distinguish bipolar patients from unipolar patients and controls (Alatiq et al., 2010) and reward sensitivity did not predict symptoms after 4 weeks in BD (Dodd et al., 2011d), yet appraisals did. Importantly, prospective associations between appraisals and outcomes were maintained when controlling for baseline symptoms and clinical variables (e.g., number of previous episodes; Dodd et al., 2011d; Lobban et al., 2013). This demonstrates the unique and specific contribution made by extreme appraisals, and suggests they are not purely an artifact of current mood symptoms or past mood episodes.

It is important to note some inconsistent findings regarding associations between extreme appraisals and mood symptoms. While some studies reported that such appraisals predicted manic, but not depressive, symptoms (Dodd et al., 2011d), others suggested an important role for these appraisals in the presence and persistence of depression, but not mania (e.g., Fletcher et al., 2014; Fisk et al., 2015). Negative appraisals of low mood appeared more pertinent for depression (e.g., Jones and Day, 2008), as would be expected.

The studies reviewed here do not suggest that any one type of appraisal is most predictive of bipolar symptoms. The ICM predicts that a range of types of extreme positive and negative appraisals would be associated with BD. For example, activated states could be appraised as likely to bring about success but also as a signal of imminent catastrophe. Consistent with this, studies that have examined relationships with distinct categories of appraisals have found that it is not only highly positive appraisals of activated states that are associated with outcomes, as would be assumed by dominant models of BD that emphasize exaggerated reward sensitivity and elevated goal pursuit (e.g., Johnson et al., 2012). In fact, across a number of studies, negative or catastrophic appraisals of activated states and mood swings were related to symptoms cross-sectionally and over time, and were elevated in bipolar groups compared to non-clinical and unipolar depressed groups (Mansell, 2006; Alatiq et al., 2010; Dodd et al., 2011a,d; Mansell et al., 2011; Lobban et al., 2013; Ruggero et al., 2015). The interaction between positive and negative appraisals has also been predictive of mania risk and BD (Kelly et al., 2011, 2016). This could explain why the overall HAPPI score is more consistently associated with outcomes than specific subscales; it may be the amalgam of different types of conflicting extreme, positive and negative appraisals that is most problematic. Normalizing appraisals of low and high mood were not elevated in those at mania risk or with BD, and were in most instances not associated with mood symptoms. This is again consistent with the ICM, which suggests that only *extreme self-referent* appraisals; i.e., where a person interprets an internal state change as being *about them*; play a role in mood swings.

Results for relationships between appraisals and outcomes differ somewhat between the appraisal measures. For example, there is evidence for longitudinal associations between outcomes and the HAPPI and BIPQ, but not the HIQ (Dodd et al., 2011d; Lobban et al., 2013). The ICM places emphasis on the combined presence of positive *and* negative appraisals of activated internal states, and the HIQ only tests one facet of this (positive self-appraisals of hypomania-relevant states). This may be why the HAPPI, measuring negative as well as positive appraisals of activated states, was significantly associated with prospective outcomes while the HIQ was not. The BIPQ assesses negative beliefs about mood swings and their consequences, and the ICM would predict that catastrophic and demoralizing beliefs (e.g., that mood swings will always result in significant symptoms and cannot be controlled through personal effort) would predict mood changes. Therefore, in future studies, the BIPQ may prove a useful tool to broaden the focus to more pervasive and long-term internal states.

Finally, evaluations of therapy are limited to one to date (Searson et al., 2012), but the finding that extreme appraisals reduce in tandem with therapy directed at them is consistent with the ICM. Taken together, these findings offer support for the ICM, which is unique in proposing that extreme negative *and* positive appraisals of both activated, manic and low, depressive internal states, play a critical role in maintaining mood swings.

### Evaluating Results in Relation to Competing Models

The research reviewed here is consistent with the ICM, but the different models of BD are complementary in some aspects and competing in others. It is therefore important to ascertain which findings are consistent with multiple alternative accounts of BD, and which are consistent only with the ICM. The aim of this section is not to review the wider literature on psychological processes in relation to different theories of BD. Rather, it is to judge whether review findings, which specifically relate to extreme positive and negative appraisals of internal states, are consistent with these theories.

Firstly, with respect to the accounts of BD based on theories of depression and depression-avoidance, studies in this review including measures of both depressogenic cognitions and extreme appraisals found independent effects for both variables, or no effect at all of dysfunctional attitudes when extreme appraisals were controlled for (e.g., Alatiq et al., 2010; Ruggero et al., 2015). This provides support for the ICM over cognitive theories of BD based on unipolar depression, because the ICM proposes extreme cognitions about internal states specifically, not dysfunctional cognitions in general, are associated with mood swings and BD.

The depression avoidance account suggests that attempts to manage negative affect and avoid depression through emotion regulation strategies such as distraction and risk-taking are associated with BD (Knowles et al., 2005). It is possible that such strategies could be prompted by negative appraisals of depressed mood states, yet this review indicates that the IDQ relates more closely to depression than to manic symptoms and mania risk (Jones and Day, 2008; Dempsey et al., 2011). Additionally, depression avoidance does not account for appraisals of (hypo)manic states, or the contradictory responses to positive affect that are also associated with mania risk and BD. These include dampening (thinking that you do not deserve to feel good) and amplifying (thinking about how happy you feel and savoring it) (Feldman et al., 2008; Gruber et al., 2009). Responses to low mood and positive affect are clearly relevant to the ICM as they can be conceptualized as descent and ascent behaviors. Findings reviewed here show that, while appraisals are associated with such response styles, appraisals predict mood symptoms and mania risk independently of these response styles (Jones and Day, 2008; Johnson and Jones, 2009; Dempsey et al., 2011; Fisk et al., 2015; Kelly et al., 2016). This emphasizes the importance of appraisals in BD, and suggests the need for research testing the relationships between appraisals, emotion regulation strategies, and outcomes.

The associations observed in some of the studies reviewed here between BD and positive appraisals of activated states, or extreme appraisals of activated states as being necessary to avoid failure or as signs of imminent personal success, are partially consistent with the circadian rhythm disruption model (Healy and Williams, 1989; Jones, 2001) and BAS dysregulation hypotheses (Urošević et al., 2008), respectively. However, unlike the ICM, neither of these theories incorporates a role for the negative appraisals of highly activated (non-depressed) mood states that were found to be associated with BD in this review. Furthermore, the measures of appraisals utilized by the studies reviewed here do not specifically refer to internal states arising from circadian rhythm or BAS disruption. The question of whether it is appraisals of internal states arising specifically from these disruptions that relate to BD remains to be tested, and so these aspects of these competing theories cannot be said to be supported by the findings reviewed here.

### Limitations

The purpose of this review was to comprehensively review research relevant to extreme appraisals of internal states and BD. The majority of the studies included were cross-sectional, and prospective studies had only short-term follow-up periods (6 months or less). Therefore firm conclusions about causal outcomes over time cannot be drawn, and more research needs to be conducted utilizing prospective designs and controlling for the correlation between extreme appraisals and concurrent mood symptoms. However, the cross-sectional studies reported in this review have not controlled for current mood because mood was frequently the outcome variable in analysis. Appraisals of internal states, mood and behavior are expected to be related to one another as part of a vicious cycle, as seen in other psychological models (e.g., Clark, 1986), and therefore correlations between appraisals and concurrent mood offer important support for the ICM. In the research reviewed here, appraisals and outcomes have primarily been assessed using self-report measures. In future, researchers should consider implicit assessments of appraisals or experimental paradigms, and utilize objective assessments of mood and behavior, to corroborate the existing literature.

Non-clinical research in analog populations was relevant to our line of enquiry, but it is important to note that much of the research reviewed here was not relating to diagnosed BD. Nonetheless, in the Introduction we highlighted important reasons for including studies of at-risk and analog samples in the review.

The findings described above support the specific predictions derived from the ICM, and unique associations have been reported between appraisals and outcomes. Nevertheless, the ICM acknowledges that a range of biopsychosocial factors contribute to the causation and maintenance of BD, including for example impulsivity, emotion regulation strategies, biological factors, and life events. Studies reviewed here have not been consistent in controlling for these important covariates, so we cannot yet draw conclusions about the extent of the contribution made by appraisals relative to other important predictors. The ICM also emphasizes the important role of behaviors and emotion regulation strategies that individuals might engage in on the basis of extreme appraisals and that might mediate their effects on mood and outcome. However, the relationship between appraisals and responses has been insufficiently studied. Finally, it is possible that dependent life events, such as the potentially traumatic nature of someone’s experiences during a mood episode and the nature of the treatment that they receive, might impact upon the way in which they later appraise internal experiences that they associate with mood episodes, and whether they appraise mood changes in extreme and negative ways. Whilst the prospective research identified in this review indicates that appraisals are not simply a cause or correlate of mood episodes and other life events, and findings from non-clinical studies bolster this argument, no studies included in this review have included direct measures of life events in order to consider their role in the development and maintenance of extreme appraisals.

### Future Directions

A number of avenues are worthy of further exploration. In particular, longer-term follow-up would enable examinations of whether extreme appraisals relate to the worsening of mood difficulties or the experience of relapse over time, and to make firmer conclusions regarding the potential causal role of appraisals in determining mood instability, mood episodes, or illness onset and course prospective designs, specifically those utilizing Experience Sampling Methodology (e.g., Solhan et al., 2009) to explore real world and dynamic, temporal associations between appraisals and outcomes, would also address the question of whether extreme appraisals are stable over time and if not what other factors predict changes in appraisals, and would also reduce recall bias. Existing research using at-risk groups could be extended using prospective methodology to examine whether extreme appraisals might predict transition from high-risk for BD, to sub-threshold, to clinical BD. This would bolster the argument that extreme appraisals are part of the diathesis for clinical mood swings, but would also inform approaches to early intervention that might lead to improved clinical outcomes.

It will also be crucial to extend existing research using experimental methods to reduce the impact of memory bias and demand characteristics on self-report data. Experimental paradigms could be utilized to investigate momentary appraisals of mood states following mood induction or in certain scenarios, enabling researchers to explore the impact of appraisals on behavior and mood in ‘real time.’

The HAPPI primarily measures appraisals of activated mood (e.g., feeling good, activated, and restless). There is little published research on the BIPQ and IDQ, yet these are the only measures of negative appraisals of mood swings more generally and low mood specifically. Clinical and longitudinal studies will be needed to examine the role that negative appraisals of low mood states play in BD.

It might be expected that cognitive mechanisms such as reasoning biases, and initial research suggests that less ability to use discomfirming evidence during a reasoning task (Haigh and Dodd, 2017). Earlier experiences stored in memory and imagery might also influence the formation and maintenance of extreme appraisals, and imagery might amplify the effect of appraisals on behavior and mood (e.g., Holmes et al., 2008). Future research could explore associations between imagery and verbal thought-based appraisals. Images and memories associated with previous traumatic episodes might be especially powerful, perpetuating the shame and self-criticism that have been associated with poor coping and emotion-regulation in BD (Lam et al., 2004; Corry et al., 2013). More research is needed to determine the relationship between trauma, shame and self-criticism and extreme appraisals of internal states, and the extent to which they might have a shared or distinct impact on bipolar spectrum mood difficulties.

While there was some evidence from the studies reviewed that extreme appraisals of internal states were predictive of bipolar symptoms over and above hypomanic personality, no studies appeared to have controlled for other differences in personality traits. There is an independent literature that documents such associations, for example with sensation-seeking (Bizzarri et al., 2007), impulsivity (Swann et al., 2001) and the ‘Big Five’ personality traits (Barnett et al., 2010). These traits may be associated with greater preponderance or persistence of certain internal states (e.g., excitement in high sensation-seeking; anxiety in high neuroticism). From the perspective of the ICM, it would be the extreme positive and negative appraisals of these states that would be more tightly linked with the escalation and maintenance of bipolar symptoms than the traits themselves. However, this awaits empirical testing.

The role played by important others in the development and maintenance of extreme appraisals relevant to BD could also be further explored, including interpersonal relationships and the family environment in particular. Interpersonal contexts may be important for the formation of certain types of appraisals. For example, individuals might develop beliefs that activated states will have negative interpersonal consequences, or restrict their autonomy, due to critical or controlling responses from others when feeling hypomanic. Alternatively, extreme appraisals might develop as a result of observing close others experiencing mood difficulties and their consequences. These experiences could also fuel appraisals encompassing shame and a negative, critical self-image. Dodd et al. (2010) extended the HAPPI measure to encompass such appraisals.

Qualitative research has contributed further to our understanding of how people appraise and manage their experiences, finding that people with BD hold ambivalent views of internal states such as positive and negative views of mania (Mansell et al., 2010; Fletcher et al., 2013). Participants valued exciting, creative aspects of mania but also wanted to avoid mania because they valued stability (e.g., “the energy can be really nice, you know, but at the back of my head there is always the worry that it might go a little too far and I’ll go manic”; Mansell et al., 2010, p. 204). People who had hypomanic experiences that resolved without causing distress, functional impairment or need for treatment (Seal et al., 2008) reported that appraising these experiences as non-problematic allowed them to engage in adaptive, mood-balancing behaviors, such as ‘going with the flow,’ or by making changes to their circumstances and environment. This research and co-production with those with lived experience should inform refinement and improve ecological validity of appraisal measures. Future research could pay careful attention to item wording to delineate between different kinds of appraisals, and recognize that emotion regulation strategies in response to appraisals may also be important predictors of outcomes.

The ICM emphasizes the role of extreme appraisals of internal states, including but not limited to mood, in the exacerbation of mood swings. Measures of appraisal differentiate those with BD from other clinical groups such as unipolar depression, but no research has explored their role in other conditions characterized by poor mood regulation, and comorbidity and mixed mood states within the context of BD.

Initial findings have indicated that appraisals interfere with social and occupational functioning over time (Dodd et al., 2011d; Lobban et al., 2013). A further important line of inquiry for future research is to test associations between extreme appraisals and further functional outcomes, such as quality of life and personal recovery (see Jones et al., 2013).

### Implications for Psychological Therapy Approaches

The results of this review indicate that extreme appraisals play a role in determining vulnerability to problematic mood swings and in maintaining them over time, suggesting that these appraisals and the beliefs that ‘feed into’ them are important targets for psychological therapy. Conflicting appraisals of internal states in particular might represent an obstacle to engagement and change in psychological therapy; some individuals may highly value (hypo)manic moods despite the distressing consequences. Clinicians may find it beneficial to identify and focus on conflicting appraisals in therapy work with these clients.

Case studies of CBT focusing on how clients’ positive, self-referent beliefs about high moods fuelled mood escalations found that clients reported improvements to their mood symptoms and changes to appraisal styles (Mansell, 2007; Mansell et al., 2007). The Think Effectively about Mood Swings (TEAMS) approach is based specifically upon this premise and prioritizes the identification and change of extreme and conflicting thoughts about mood swings (Mansell, 2007). So far this has only been evaluated in a case series (Searson et al., 2012); the first randomized controlled trial of TEAMS is ongoing (Mansell et al., 2014).

The finding that extreme appraisals of internal states play a role in determining mood symptoms also has potential implications for caregivers and professionals, as it may be important to consider their potential role in inadvertently confirming or driving extreme appraisals, through the advice they give and their own responses. For example, advising someone to be vigilant for early signs of relapse might fuel their belief that minor fluctuations in mood are markers of an imminent breakdown. Clinicians have speculated that hypervigilance to mood shifts might lead to inappropriate safety behaviors and avoidance (e.g., Schwannauer, 2004); this is yet to be tested empirically.

Qualitative research has reported that appraisals of internal states not only influenced attempts to regulate these states, but medication use (Cappleman et al., 2015), and suggests that individuals find it helpful to adopt a less judgemental, present-focused approach to mood changes (Seal et al., 2008). This is consistent with mindfulness-based approaches, which have potential utility within BD (Miklowitz et al., 2009). By practicing non-judgemental awareness of the present moment, individuals can increase awareness of mood fluctuations without appraising these fluctuations in extreme ways or engaging automatically in emotion regulation strategies that lead to further mood changes.

## Conclusion

This review has identified converging evidence suggesting that extreme appraisals are (1) a risk or cognitive vulnerability factor for the development of bipolar (appraisals are elevated in BD and associated with mania risk in undiagnosed samples) and (2) a risk factor for future episodes and diminished functioning (appraisals are associated with symptoms and poorer functioning both cross-sectionally and prospectively), as predicted by the ICM (Mansell et al., 2007). Extreme appraisals were consistently elevated in bipolar relative to non-clinical controls and other clinical groups and were associated with bipolar-relevant outcomes in non-clinical studies. This suggests that extreme appraisals are associated with mood difficulties across the bipolar continuum; extreme appraisals are endorsed by individuals experiencing mood symptoms but without a diagnosis of BD, but these appraisals are likely to become more extreme, self-fulfilling, and problematic among those individuals with a history of clinically significant mood episodes. Nevertheless, appraisals are predictive of outcomes over and above illness course and past experiences; appraisals are associated with a diagnosis of BD even when controlling for current mood, and are associated with mania risk and subclinical symptomatology in non-clinical groups who have not experienced clinical mood episodes.

The ICM and alternative psychological models of BD are not mutually exclusive. Nonetheless, the review has identified that both positive and negative extreme appraisals of different internal states are associated with outcomes across the bipolar spectrum, consistent with the ICM but not predicted by other psychological theories of BD. It is clear that the cognitions implicated in the ICM are important in BD, and this has implications for therapeutic work with individuals seeking help for problematic mood swings.

## Author Contributions

RK and AD conducted the systematic literature review. RK and AD wrote the first draft and WM provided critical review. Each of the authors contributed to and approved the final draft.

## Conflict of Interest Statement

The authors declare that the research was conducted in the absence of any commercial or financial relationships that could be construed as a potential conflict of interest.
