# Supplementary material for: “When my Moods Drive Upward There Is Nothing I Can Do about It”: A Review of Extreme Appraisals of Internal States and the Bipolar Spectrum
Source: Front Psychol. 2017 Aug 4;8:1235. doi: 10.3389/fpsyg.2017.01235 (PMC5543079; doi:10.3389/fpsyg.2017.01235)
Supplement: Supplementary file 1 [file Table_1.docx]

**Table 1: Studies in non-clinical groups**

| Author(s) (date) | Population (*n*) | Design/method | Measures | Key findings | |
| --- | --- | --- | --- | --- | --- |
|  |  |  |  | Hypothesis 1 | Hypotheses 3/4 |
| Ankers & Jones (2009)* | Non-clinical student sample  High risk (n = 31)  Controls (n = 24) | Cross-sectional group comparison    *Included a prospective diary study that did not explore role of appraisals* | *Appraisals:*  HIQ  *Mania risk:*  Hypomanic personality (HPS)  *Mood symptoms:*  Activation, depression, conflict and well-being (Internal States Scale; ISS) | High risk > controls on positive self-appraisals of hypomania  No group difference on normalising appraisals of hypomania  Positive self-appraisals predicted group membership (high risk vs. control) when controlling for sleep variables and mood | --- |
| Dempsey, Gooding & Jones (2011) | Non-clinical students (n = 353) | Cross-sectional correlational | *Appraisals:*  HIQ  IDQ  *Emotion regulation strategies:*  Responses to positive affect (RPA; Feldman, Joorman & Johnson, 2008)  Ruminative response styles (RRS; Nolen-Hoeksema & Morrow, 1993)  *Mania risk:*  HPS  *Symptoms:*  Centre for Epidemiologic Studies – Depression (CES-D; Radloff, 1977)  Activation, depression, conflict and well-being (ISS)  Hypomania in past 3 months (HIQ-Exp)  Depression in past 3 months (IDQ-Exp) | Positive self-appraisals of hypomania were significantly associated (+ve ) with mania risk while controlling for current mood, negative appraisals of depression, recent experience of hypomania and depression, and emotion regulation strategies  No associations normalising appraisals and mania risk  Principal components analysis (PCA) found variable ‘positive cognitive style’ (including HIQ-H items) which predicted mania risk, when controlling for mood | Negative self-appraisals of depression significantly associated with depressive symptoms while controlling for current mood, recent experience of hypomania and depression, mania risk, normalising appraisals, dampening, self-focused rumination, and ruminative response styles  No association between normalising appraisals and depressive symptoms  PCA found ‘negative cognitive style’ including appraisals which predicted higher depressive symptoms and ‘positive cognitive styles’ which predicted fewer depressive symptoms, when controlling for mood |
| Dodd & Haigh (in press) | Non-clinical community sample  *Study 1*  n = 150  *Study 2*  n = 241 | Cross-sectional correlational | *Appraisals:*  HAPPI  *Mood symptoms:*  Activation (ISS) | --- | Extreme appraisals positively associated with activation (both studies) |
| Dodd, Mansell, Sadhnani, Bentall & Tai (2010) | Non-clinical students (n = 134) | Longitudinal (3 month follow up) correlational | *Appraisals:*  HAPPI (61 items)  *Other cognitive styles:*  Reward sensitivity (BIS/BAS; Carver & White, 1994)  Dysfunctional attitudes (DAS; Weissman & Beck, 1978)  *Mania risk:*  HPS  *Symptoms:*  Mania in 3 month follow up period (adapted MDQ)  Activation, depression, conflict and well-being (ISS) | --- | Controlling for mood, appraisals were significantly associated with:   - Manic symptoms (+ve) - Activation (+ve) - Well-being (-ve)   Association with Activation remained significant when controlling for age, gender, mania risk, reward sensitivity and dysfunctional attitudes  Appraisals did not predict depressive symptoms or conflict |
| Dodd, Mansell, Morrison & Tai (2011a) | Non-clinical students (n = 253) | Cross-sectional correlational | *Appraisals:*  HAPPI (61 items) – subscales   - Social Self-Criticism - Increasing Activation to Avoid Failure - Success Activation & Triumph Over Fear - Regaining Autonomy - Loss of Control - Grandiose Appraisals of Ideation   *Symptoms:*  Activation, depression, conflict and well-being (ISS) | ---- | Social Self-Criticism associated (+ve) with activation, conflict and depressive symptoms  Increasing Activation to Avoid Failure associated with conflict and depressive symptoms (+ve), well-being -ve  Success Activation & Triumph Over Fear associated with well-being (+ve)  All other associations with subscales and symptoms non-significant |
| Dodd, Mansell, Bentall & Tai (2011b) | Non-clinical students (n = 175) | Prospective (4 day diary) | *Appraisals:*  HAPPI (61 items)  *Other cognitive styles:*  Reward sensitivity (BIS/BAS)  *Emotion regulation strategies:*  Ascent & descent behaviours (Behaviours Checklist; Fisk, Dodd & Collins, 2015)  *Mania risk:*  HPS  *Symptoms:*  Activation, depression, conflict and well-being (ISS) | --- | While controlling for mood, age, gender, mania risk, reward sensitivity, appraisals were significantly associated with (+ve):   - Activation - Conflict - Depressive symptoms - Ascent Behaviours   Appraisals not associated with well-being |
| Dodd, Mansell, Morrison & Tai (2011c) | Student sample  Bipolar risk (n = 18)  Depression risk (n = 20)  Controls (n = 30) | Cross-sectional group comparison | *Appraisals:*  Computerised ratings task (Internal State Appraisals Task)  *Symptoms:*  Computerised mood rating | Bipolar risk > controls on catastrophic appraisals of low activation, when controlling for mood  Depression risk > controls on negative appraisals, when controlling for mood | --- |
| Dodd et al (2013)* | Student samples  *Study 1*  Cognitive high-risk defined by high HAPPI score (n = 32)  Controls defined by low/mid HAPPI score (n = 32)  *Study 2*  Cognitive high-risk defined by high HAPPI score (n = 15)  Controls defined by low/mid HAPPI score (n = 15) | Cross-sectional group comparison | *Appraisals:*  HAPPI (61 items)  HIQ  *Other cognitive styles:*  Reward sensitivity (BIS/BAS)  Dysfunctional Attitudes (DAS)  *Mania risk:*  HPS  *Symptoms:*  Observer-rated behavioural symptoms (pressure of speech)  History of manic symptoms (MDQ)  Recent hypomanic symptoms (HIQ-Exp)  Activation, depression, conflict and well-being (ISS) | *Study 1*  Cognitive high-risk > controls on mania risk and history of manic symptoms  *NB. Significant differences also on Dysfunctional Attitudes, BAS Reward Responsiveness, HIQ-Normalising but not HIQ-Hypomania* | *Study 1*  Cognitive high-risk > controls on:   - Activation - Depressive symptoms - Pressure of speech   When controlling for normalising appraisals and dysfunctional attitudes, only significant difference that remained was on depressive symptoms  When controlling for reward dysregulation and sensitivity, significant differences remained on activation and depressive symptoms  *Study 2*  Cognitive high-risk > controls on:   - Activation - Depressive symptoms - Conflict   No group differences on well-being |
| Espie, Jones, Vance & Tai (2012) | Adolescent children of a parent with BD (CPB, n = 23)  Adolescent controls (n = 24) | Cross-sectional group comparison | *Appraisals:*  HIQ  *Other cognitive styles:*  Attributional styles (ASQ; Peterson et al, 1982)  *Mania risk:*  HPS | No difference between CPB and controls on positive appraisals of hypomania  In entire sample, positive appraisals of hypomania predicted mania risk, while controlling for familial risk and internality for positive events | --- |
| Fisk, Dodd & Collins (2015) | Non-clinical (n = 134) | Cross-sectional correlational | *Appraisals:*  HAPPI (29-item)  *Mania risk:*  HPS  *Emotion regulation strategies:*  Response styles to positive and negative affect (RPA and RSQ)  Ascent and descent behaviours (BC)  *Symptoms:*  CES-D  Altman Scale for Rating Mania (ASRM; Altman et al, 1997) | HAPPI significantly associated (+ve) with mania risk when controlling for mood  Association with mania risk remained significant while controlling for response styles (dangerous activities, dampening, emotion-focused and self-focused rumination, ascent behaviours) | HAPPI associated (+ve) with depressive symptoms  HAPPI no longer significantly associated with depressive symptoms when controlling for age, mania risk, and response styles  HAPPI not associated with manic symptoms |
| Haigh & Dodd (2017) | Non-clinical students (n = 150) | Cross-sectional correlational | *Appraisals:*  HAPPI  *Mania risk:*  HPS  *Symptoms:*  Activation, depression, conflict and well-being (ISS) |  | HAPPI positively associated with:   - Activation (+ve) - Depression (+ve) - Conflict (+ve) - Mania risk (+ve)   Association between HAPPI and mania risk sustained when controlling for age, symptoms and reasoning bias  *Analyses with HAPPI subscales:*  Activation associated with all factors (+ve)  Conflict and depressive symptoms associated with Increasing Activation, Loss of Control, Regaining autonomy, Social Self-Criticism (+ve)  Well-being associated with Increasing Activation, Social Self-criticism (-ve) and Success Activation (+ve) |
| Johnson & Jones (2009) | Non-clinical students - UK and US (n = 638) | Cross-sectional correlational | *Appraisals:*  HIQ  *Other cognitive styles:*  Positive Overgeneralisation  *Emotion regulation strategies:*  Barratt Impulsivity Scale (BIS; Patton & Stanford, 1995)  Responses to positive affect (RPA)  *Mania risk:*  HPS  *Symptoms:*  HIQ-Experience | Positive appraisal items from HIQ separable factor from other variables in PCA and this factor independently associated with mania risk when controlling for recent experience of hypomania, country of residence, gender, overconfidence and response styles | --- |
| Jones & Day (2008) | Non-clinical university population (n = 231) | Cross-sectional correlational | *Appraisals:*  HIQ  IDQ  *Other cognitive styles:*  Threat/reward sensitivity (BIS/BAS)  Dysfunctional Attitudes (DAS)  *Mania risk:*  HPS  *Symptoms:*  Activation, depression, conflict and well-being (ISS)  Depression (CES-D) | Positive appraisals of hypomania predicted hypomanic personality when controlling for symptoms and other cognitive styles | Negative self-appraisals of depression-relevant experiences predicted depressive symptoms, controlling for other symptoms and cognitive styles |
| Jones, Mansell & Waller (2006) – Study 1* | Non-clinical college students (n = 203) | Cross-sectional group comparison | *Appraisals:*  HIQ  *Other cognitive styles:*  Dysfunctional Attitudes (DAS)  *Mania risk:*  HPS  *Symptoms:*  Recent experience of hypomania (HIQ-Experience) | Positive appraisals of hypomania-relevant experiences associated (+ve) with mania risk when controlling for recent experience of hypomania | --- |
| Kelly et al (2012) | Non-clinical (n = 323) | Cross-sectional correlational | *Appraisals:*  HAPPI (50 item version - split into positive appraisals and negative appraisals)  *Symptoms:*  Activation and depression (ISS) | --- | Positive appraisals associated (+ve) with activation when controlling for depressive symptoms and gender  Negative appraisals associated (+ve) with depressive symptoms when controlling for activation and gender |
| Kelly et al (2016) | Non-clinical adolescent (n = 98) | Cross-sectional correlational | *Appraisals:*  HAPPI (adapted for use in adolescents aged 13-17)  *Mania risk:*  MDQ  *Symptoms:*  Activation and depression (ISS)  Irritability  *Emotion regulation strategies:*  RPA-Child version  Impulsivity (BAS Fun-seeking) | Interaction between positive and negative appraisals predicted being in high or moderate risk group. | Positive appraisals associated (+ve) with activation when controlling for depressive symptoms and gender  Negative appraisals associated (+ve) with depressive symptoms and irritability when controlling for other symptoms and gender  When controlling for responses to positive affect and impulsivity, HAPPI was associated (+ve) with activation, depressive symptoms, irritability, and history of manic symptoms |
| Mansell, Rigby, Tai & Lowe (2008) | Non-clinical students (n = 191) | Cross-sectional correlational | *Appraisals:*  HAPPI (50 items) – subscales   - Success activation & triumph over fear - Activating response style - Loss of control when activated - Reduced social regulation - Catastrophic beliefs of internal states   *Other cognitive styles:*  Threat/reward sensitivity (BIS/BAS)  *Mania risk:*  HPS  *Symptoms:*  Lifetime experience of mania (MDQ)  Activation, conflict, depression and well-being (ISS) | --- | HAPPI predicted past hypomania when controlling for threat/reward sensitivity and mania risk  *Analyses with HAPPI subscales (controlling for age, sex, threat/reward sensitivity and mania risk):*  Success Activation predicted (+ve) well-being  Catastrophic predicted (-ve) well-being  All factors associated with activation (all +ve except Catastrophic)  Conflict associated (+ve) with Reduced Social Regulation, Loss of Control, Catastrophic  Depressive symptoms -ve associated with Success Activation, +ve associated with Reduced Social Regulation and Catastrophic beliefs |
| Tosun et al (2015) | Community sample (n = 103)  *This study also included a bipolar group as reported in Table 2* | Cross-sectional group comparison and correlational study | *Appraisals:*  Brief-HAPPI (Turkish version)  *Other cognitive styles:*  DAS  *Symptoms:*  MDQ  Case notes for clinical variables | Number of previous hypomanic symptoms (control group) predicted by HAPPI but not DAS | --- |

--- Not tested in this study * *Other psychological process variables were measured but analysed separately from appraisal measures so are not reported here*

**Table 2: Studies in clinical groups**

| Author(s) (date) | Sample | Design/methods | Appraisal measure and other measures | Key findings | | |
| --- | --- | --- | --- | --- | --- | --- |
|  |  |  |  | Hypothesis 1 | Hypothesis 2 | Hypotheses 3/4 |
| Alatiq et al (2010) | BD (n = 40)  Unipolar (n = 20)  Community sample (n = 20) | Cross-sectional group comparison | *Appraisals:*  HAPPI (50 items)  *Other cognitive styles:*  Dysfunctional Attitudes (DAS)  *Symptoms:*  Depression (Hamilton Depression Scale)  Mania (Young Mania Rating Scale) | --- | BD group > unipolar group and controls on HAPPI, when controlling for mood  BD group > controls on Self Catastrophic subscale  BD group > both groups on Other Negative and Response Styles subscales | --- |
| Dodd, Mansell, Morrison & Tai (2011d) | BD (n = 50) | Longitudinal (4 week follow up) | *Appraisals:*  HAPPI (61 items) – subscales   - Social Self-Criticism - Increasing Activation to Avoid Failure - Success Activation & Triumph Over Fear - Regaining Autonomy - Loss of Control - Grandiose Appraisals of Ideation   HIQ  *Other cognitive styles:*  Threat/reward sensitivity (BIS/BAS)  *Symptoms/functioning:*  Activation, conflict, depression and well-being (ISS)  Recent experience of hypomania (HIQ-Exp)  Functioning (Work & Social Adjustment Scale; WSAS) | --- | --- | HAPPI predicted activation and conflict (+ve) plus functioning (-ve) when controlling for baseline symptoms and functioning  Association with activation remained significant when controlling for number of months since last episode (mania and depression)  Association with conflict remained significant when controlling for recent experience of hypomania, months since last episode of depression, number of hours of CBT in lifetime  HAPPI did not predict depressive symptoms or well-being  *Analyses using HAPPI subscales:*  Increasing Activation to Avoid Failure associated with activation  Success Activation associated (-ve) with depression and functioning  Loss of Control associated with depressive symptoms (+ve) and functioning (-ve)  HIQ-H not associated with prospective symptoms and functioning |
| Fletcher, Parker & Manicavasagar (2014)* | Adults BD (n = 151)  BD I (n = 69)  BD II (n = 82) | Longitudinal (6 month follow up) | *Appraisals:*  HAPPI (50 item)  *Symptoms/mood:*  Observer-rated mood episodes (MINI International Neuropsychiatric Interview)  Activation and depression (ISS)  Anxiety (Spielberger Anxiety Inventory-State) | --- | --- | HAPPI associated with variability in depressive symptoms over 6 month follow up in BD I and BD II (+ve) when controlling for baseline symptoms and age  HAPPI not associated with severity of activation or depression or variability in activation over 6 month follow up  HAPPI did not predict observer-rated mood episodes (mania and depression) at 6 months |
| Jones, Mansell & Waller (2006) – Study 2 | BD (n = 56)  Non-clinical (n = 39) | Cross-sectional group comparison | *Appraisals:*  HIQ  *Mania risk:*  HPS  *Symptoms:*  Activation, conflict, depression, well-being (ISS) | --- | BD group > controls on positive appraisals of hypomania, when controlling for symptoms | --- |
| Kelly, Mansell, Sadhnani & Wood (2011) | BD (n = 171)  Depression (n = 42)  Controls (n = 64) | Cross-sectional group comparison | *Appraisals:*  HAPPI (50 item) - split into positive appraisals and negative appraisals) | --- | Interaction between positive and negative appraisals predicted BD diagnosis | --- |
| Lobban et al (2013)* | BD (n = 91) | Longitudinal (24 week follow up) | *Appraisals:*  Brief Illness Perception Questionnaire  *Symptoms/functioning:*  Depression (Hamilton Rating Scale for Depression; HAM-D)  Mania (Bech-Rafaelson Mania Rating Scale; BMRS)  Observer-rated mania and depression (SCID-LIFE)  Social and Occupational Functioning Assessment Scale (SOFAS) | --- | --- | Less control over mood swings, and less perceived personal effort in managing mood swings associated with current depressive symptoms (+ve)  Beliefs about negative consequences of mood swings associated with functioning (-ve)  Beliefs about negative consequences of mood swings, their role in personal identity, and concern about mood swings predicted time to depressive relapse over 24 weeks, when controlling for baseline symptoms  Beliefs about negative consequences of mood swings associated with more fluctuations in depressive symptoms  Perceived personal effort in overcoming mood swings associated with less fluctuation in depressive symptoms over 24 weeks  Beliefs about mood swings were not associated with current manic symptoms, fluctuations in manic symptoms, or manic relapse  Longitudinal associations were significant when controlling for baseline symptoms |
| Mackali et al (2014)* | BD (n = 115)  Community sample (n = 103) | Cross-sectional | *Appraisals:*  Brief-HAPPI (Turkish)  *Symptoms:*  Lifetime history of mania (MDQ) | --- | BD group > controls on appraisals of internal states | HAPPI correlated with past experience of manic symptoms |
| Mansell (2006) | BD (n = 22)  Non-clinical (n = 22) | Cross-sectional group comparison | *Appraisals:*  HAPPI (50 items) – subscales   - Self-activation - Response style - Other-positive - Other-negative - Self-catastrophic   *Mood:*  Self-reported mood and energy level (VAS) |  | BD > control group on HAPPI and all subscales, when controlling for mood |  |
| Mansell & Jones (2006) | BD (n = 56)  University sample (n = 39) | Cross-sectional group comparison | *Appraisals:*  Brief-HAPPI  HIQ  *Symptoms:*  Activation, conflict, depression and well-being (ISS) |  | Appraisals of internal states and positive appraisals of hypomanic experiences predicted BD diagnosis, when controlling for symptoms |  |
| Mansell et al (2011)* | BD (n = 30)   - BD relapsed (past 2 years; n = 16) - BD recovered (n = 14)   Unipolar depression (n = 22)  Non-clinical (n = 22)  History of hypomania only (n = 16) | Cross-sectional group comparison | *Appraisals:*  HAPPI (50 items) – subscales   - Success activation & triumph over fear - Activating response style - Loss of control when activated - Reduced social regulation - Catastrophic beliefs of internal states   *Symptoms:*  Activation, conflict, depression and well-being (ISS) |  | Bipolar relapsed and overall BD group (including recovered) > unipolar group and non-clinical controls but not hypomania only group on HAPPI, when controlling for symptoms  Unipolar group > non-clinical group on HAPPI  *Analyses using HAPPI subscales:*  Relapsed BD > unipolar group on Success Activation, Reduced Social Regulation, Loss of Control  Relapsed BD > hypomania only group on Catastrophic  Relapsed BD group > non-clinical group on all subscales  Recovered BD and hypomania only groups > non-clinical group on Success Activation and Loss of Control |  |
| Palmier-Claus, Dodd, Tai, Emsley & Mansell (2015) | BD (n = 52) | Prospective | *Appraisals:*  HAPPI (split by positive and negative appraisals)  *Emotion regulation:*  Ascent/descent behaviours  *Symptoms:*  Activation and depression (ISS) |  |  | Positive appraisals directly related to subsequent activation (+ve) when controlling for baseline symptoms. This was partially mediated by behaviours. |
| Pavlickova et al (2014)* | CPB (13-19 years, n = 30)  Controls (n = 30) | Cross-sectional group comparison | *Appraisals:*  HAPPI (29-item) | No difference between CPB and control children on HAPPI | Parents with BD > parents without BD on HAPPI  Affected CPB (any diagnosis) > non-affected CPB and controls on HAPPI |  |
| Ruggero, Bain, Smith & Kilmer (2015)* | Adult CPB (n =27)  Adult offspring of parents with depression (n = 30)  Adult offspring of parents no mood disorder (n = 32) | Cross-sectional group comparison | *Appraisals:*  HAPPI (61 items) - subscales as per Dodd et al (2011d) | Adult CPB and adult offspring of parents with depression > adult offspring of parents with no mood disorder on HAPPI | Those diagnosed with BD > those with depression and controls on HAPPI subscales.  BD diagnosis predicted Increasing Activation to Avoid Failure, Social Self-criticism, Loss of Control, Regaining Autonomy |  |
| Tosun et al (2015) | Remitted bipolar (n = 118)  Community sample (n = 103) | Cross-sectional group comparison and correlational study | *Appraisals:*  Brief-HAPPI (Turkish version)  *Other cognitive styles:*  DAS  *Symptoms:*  MDQ  Clinica case notes | --- | Bipolar > controls on HAPPI  HAPPI predicted BD diagnosis in the same model as DAS and MDQ | Length of remission –ve associated with HAPPI (bipolar group)  No significant differences on HAPPI for those who had experienced (hypo)mania only and those who had also had depressive/mixed mood |

--- Not tested in this study * *Other psychological process variables were measured but analysed separately from appraisal measures so are not reported here*

NB: Diagnosis confirmed via structured clinical interview in all studies with clinical groups or offspring of clinical groups (except Mansell, 2006; Jones, Mansell & Waller, 2006; Tosun et al, 2015)
